# Neural and Endocrine Correlates of Early Life Abuse in Youth With Depression and Obesity

**DOI:** 10.3389/fpsyt.2018.00721

**Published:** 2018-12-21

**Authors:** Kevin L. Sun, Kathleen T. Watson, Sarthak Angal, Baylee F. Bakkila, Aaron J. Gorelik, Sara M. Leslie, Natalie L. Rasgon, Manpreet K. Singh

**Affiliations:** Department of Psychiatry and Behavioral Sciences, Stanford University School of Medicine, Stanford, CA, United States

**Keywords:** pediatric depression, obesity, insulin resistance, early life stress, abuse, resting state functional connectivity, diabetes

## Abstract

Depression and insulin resistance are becoming increasingly prevalent in younger populations. The origin and consequence of insulin resistance in depressed youth may, in part, be rooted in exposure to environmental stressors, such as early life abuse, that may lead to aberrant brain motivational networks mediating maladaptive food-seeking behaviors and insipient insulin resistance. In this paper, we aimed to investigate the impact of early life abuse on the development of insulin resistance in depressed and overweight youth aged 9 to 17 years. We hypothesized that youth with the greatest burden of early life abuse would have the highest levels of insulin resistance and corresponding aberrant reward network connectivities. To test this hypothesis, we evaluated sixty-nine depressed and overweight youth aged 9 to 17, using multimodal assessments of early life abuse, food-seeking behavior, and insulin resistance. Based on results of the Childhood Trauma Questionnaire (CTQ), we separated our study participants into two groups: 35 youth who reported high levels of the sum of emotional, physical, or sexual abuse and 34 youth who reported insignificant or no levels of any abuse. Results of an oral glucose tolerance test (OGTT) and resting state functional connectivity (RSFC), using the amygdala, insula, and nucleus accumbens (NAcc) as seed-based reward network regions of interest, were analyzed for group differences between high abuse and low abuse groups. High abuse youth exhibited differences from low abuse youth in amygdala-precuneus, NAcc-paracingulate gyrus, and NAcc-prefrontal cortex connectivities, that correlated with levels of abuse experienced. The more different their connectivity from of that of low abuse youth, the higher were their fasting glucose and glucose at OGTT endpoint. Importantly, level of abuse moderated the relation between reward network connectivity and OGTT glucose response. In contrast, low abuse youth showed hyperinsulinemia and more insulin resistance than high abuse youth, and their higher OGTT insulin areas under the curve correlated with more negative insula-precuneus connectivity. Our findings suggest distinct neural and endocrine profiles of youth with depression and obesity based on their histories of early life abuse.

## Introduction

Depression is a common psychiatric disorder that significantly impacts youth worldwide (1, 2). In parallel, rates of obesity among youth are also rising, resulting in the common co-occurrence of these conditions that may lead to an increased risk of cardiovascular disease, type 2 diabetes mellitus, and all-cause mortality (3, 4). Indeed, multiple studies have demonstrated a clear relation between depression and insulin resistance (5), which is the precursor to diabetes when cells fail to respond to the normal actions of insulin (6–8). Specifically, a bidirectional relation between depressive symptoms and diabetes is evident, in which the presence of one increases the incidence and severity of the other (9). In pediatric populations, this bidirectional relation has, in part, been linked to a dysregulated stress system (10).

Stress exposure in early childhood arises in different types of adverse childhood experiences, each with unique consequences on brain and biological development (11). Indeed, recent studies distinguish the effects of inadequate input (such as neglect and deprivation) from the effects of harmful input (such as abuse and trauma) on brain structure and function, as well as on psychopathology, cortisol metabolism, and epigenetic changes (12–14). Among these various maltreatment inputs, childhood abuse has been shown to worsen depression course (15), and emotional abuse in particular has been shown to best predict psychiatric symptomatology in children compared to other maltreatment types (16). Importantly, childhood abuse can increase the risk of developing insulin resistance in adulthood (17, 18). Contributing to this literature, we here focus principally on exposure to childhood abuse and its specific neural and endocrine correlates.

Independent studies in pediatric populations affected by depression, insulin resistance, and childhood abuse converge on impairment in a common reward network. This mesolimbic reward circuit consists primarily of the prefrontal cortex (PFC), nucleus accumbens (NAcc), ventral tegmental area (VTA), amygdala, and hippocampus (19). In depression, a dysregulated reward network can lead to reward hyposensitivity and contribute to the motivational deficits of anhedonia (20). With regards to insulin resistance, multiple insulin receptors are found in key reward centers of the brain (21, 22), exerting their central actions through aiding the release of dopamine (23–25) and the inhibition of GABA (26). Youth with insulin resistance show dysfunction in limbic and striatal subregions of this reward network (27–29). Finally, several studies suggest that youth with early childhood adversity experience dysfunction of the prefrontal-limbic (30, 31) and basal ganglia portions of the reward network leading to blunted reward responses (32, 33).

Previous studies that investigated different combinations of depression, insulin resistance, and childhood abuse also implicated dysregulation of the mesolimbic reward network (34–36). Our prior study presented a potential neural link between depression and insulin resistance through dysregulation of the neural reward network, as overweight youth with depression and insulin resistance had higher levels of dysconnectivity between the anterior cingulate cortex and hippocampus, as well as reduced volumes of these regions (37). It is unknown whether childhood abuse mediates altered regulation of the neural reward network in these youth, leading to the potential for either resilient or lifelong depression and diabetes outcomes.

In this study, we evaluated 69 children between the ages of 9 to 17 with depression and a BMI at or above the 85th percentile. We hypothesized that higher levels of early childhood abuse would be associated with higher levels of insulin resistance, as demonstrated by higher fasting insulin levels, higher fasting glucose levels, and a persistently elevated glucose curve after an oral glucose tolerance test (OGTT) (38, 39). We also hypothesized that youth with depression and obesity who had higher compared to lower levels of abuse would have more dysfunctional connectivity in the reward neural network. We selected the amygdala, insula, and nucleus accumbens (NAcc) as regions of interest due to their central roles in the reward network common to depression, insulin resistance, and childhood abuse literatures (21, 22, 30).

## Materials and Methods

### Study Participants, Screening Procedures, and Analytic Approach to Behavioral Correlates of Insulin Resistance

Sixty-nine overweight or obese youth between the ages of 9 to 17 years with currently untreated depressive symptoms were recruited for this study. All participants provided written assent and at least one parent or legal guardian provided written informed consent prior to all study procedures. This study was approved by Stanford University's Institutional Review Board. Participants were recruited from pediatric mood and weight control programs and community advertisements.

Youth were included if their body mass index (BMI) was at the 85th percentile or higher for their age and sex based on the Center for Disease Control and Prevention BMI calculator for children and teens (https://nccd.cdc.gov/dnpabmi/calculator.aspx). During the screening visit, height (with accuracy of 0.1 cm) and weight (with accuracy of 0.1 kg) were measured with the Seca 284, an electronic measuring station, after the removal of shoes and jackets. Two measures of height were obtained and averaged. We assessed Tanner stage using the youth self-reported Pubertal Development Scale (40) in conjunction with a clinician's physical examination of secondary sex characteristics to confirm the self-reported rating. Participants were also evaluated on levels of depression severity using the Children's Depression Rating Scale-Revised (CDRS-R) (41) administered separately to parents and youth by a study clinician or trained coordinator. Participants were included if their raw CDRS-R summary scores were greater than 35, signifying at least moderate levels of depression severity at the time of enrollment. All youth in this sample were treatment-seeking for active unremitted symptoms, but unmedicated, and were generally early in the course of their depressive illness.

Individuals were assessed for current and lifetime psychiatric disorders with the Kiddie Schedule for Affective Disorders and Schizophrenia-Present and Lifetime version (KSADS-PL) (42). Youth were excluded if they were already being treated for a mood disorder when evaluated at the screening visit. Youth were also excluded if they had type 1 or type 2 diabetes, were taking medication that affected their mood, weight, or metabolism at the time of screening, had a contraindication for an MRI (e.g., metal in their body or anterior-posterior diameter > 46 cm), or if their Full-4 IQ score on the Wechsler Abbreviated Scale of Intelligence (WASI) (43) was <70.

The Childhood Trauma Questionnaire (CTQ) was used as our primary clinical assessment of early childhood abuse. The CTQ is a 28-item scale of childhood trauma self-reported by youth participants (44). The CTQ separates trauma into five categorical scores with five questions per category: emotional abuse, physical abuse, sexual abuse, emotional neglect, and physical neglect. We secondarily used the Multidimensional Peer Victimization Scale (PVS) to understand other environmental forms of abuse, such as bullying, that are commonly experienced by youth outside of the home (45). This scale is a 16-item self-report measure with 4 subscales, to assess physical victimization, verbal victimization, social manipulation, and property attacks. We used a totaled PVS score in our linear models to adjust for the possible confounding effect of peer victimization on the relation between abuse and neural and endocrine outcomes, as childhood bullying has been shown to predict systemic inflammation and being overweight in early adulthood (46, 47). We used the Three-Factor Eating Questionnaire (TFEQ) (48) in youth to contexualize neural findings with behavioral measures of food-seeking behavior, including the constructs of cognitive restraint, uncontrolled eating or eating disinhibition, and emotional eating. Finally, we assessed socioeconomic status by the Hollingshead Four-Factor Index of Social Status and reported participants' raw scores (49).

### Assessment of Insulin Resistance

After the screening visit, eligible youth were assessed for insulin resistance. Serum markers of insulin resistance were assessed using a 2-h oral glucose tolerance test (OGTT). After a 10-h fasting period and an initial fasting blood draw, participants consumed 75 g of oral glucose and had their blood drawn every 30 min for 2 h to measure insulin and glucose (37). Insulin values were measured by immunoassay.

Insulin response to OGTT was determined by calculating the area under the serum insulin curves, plotted at each time point during the OGTT. Graphing plasma glucose levels vs. time and finding the area under the curve similarly calculated glucose response to OGTT. Insulin resistance was determined by fasting insulin measure and by HOMA-IR, which was calculated using the equation fasting insulin (mU/mL) × fasting glucose (mg/dL)/405 (50).

### Childhood Trauma Questionnaire (CTQ) Groupings

Using the cumulative CTQ categories of emotional abuse, physical abuse, and sexual abuse, we grouped participants into “high abuse” and “low abuse” groups. Here, we focused on abuse rather than emotional or physical neglect, due to previous findings of childhood abuse as risk factors for metabolic outcomes such as prediabetes and diabetes (51, 52), and due to the demonstrated role of emotional abuse in predicting psychopathology (16). We classified participants as highly abused if they met the threshold for “low to moderate” abuse, as defined by the CTQ manual, for at least one of the three aforementioned categories. We used the following “high abuse” cutoff scores: an emotional abuse categorical score > 9; a physical abuse categorical score > 8; and a sexual abuse categorical score > 6. We also totaled the three abuse scores for a total CTQ abuse score that we used as a continuous variable in linear modeling. In order to be included in the “low abuse” group, youth could not endorse abuse above any of the cut-off scores for the three CTQ categories of abuse. Using such criteria, we were able to define with confidence a “low abuse” group of youth who did not report any significant early life stress from childhood abuse.

### Neuroimaging Data Acquisition

We used neuroimaging to investigate neural connectivity group differences at resting state, assessing for neural markers of approach motivation. Using functional MRI data of a subset of our participants (*n* = 47), we seeded three different regions that have been implicated in reward and depression: amygdala, insula, and nucleus accumbens (NAcc). After participants were familiarized with the scanning environment in an MRI simulator, whole-brain images were acquired on a 3T GE Signa Excite (General Electric Co., Milwaukee, WI) scanner equipped with an 8-channel head coil. Functional images were collected at rest using a spiral pulse sequence with the following parameters: repetition time (TR) = 2,000 ms, echo time (TE) = 30 ms, flip angle (FA) = 80°, field of view (FOV) = 22 cm, number of slices = 30 slices in the axial plane, and slice thickness = 4 mm with a gap of 1 mm. The first four volumes of each resting state scan were discarded at the scanner to allow for stabilization of longitudinal magnetization. High-order shimming was used before acquisition of resting state data to improve field inhomogeneity. High-resolution structural images were also collected to assist in registration of functional data to standard space using a fast spoiled gradient recalled (3D FSPGR) pulse sequence with the following parameters: TR = 8.5 ms, TE = 3.32 ms, TI = 400 ms, flip angle = 15°, field of view(x) = 25.6 cm, matrix of 256 × 256, number of slices = 186 slices in the axial plane, and a slice thickness of 1 mm.

### Functional MRI Pre-Processing

Pre-processing of resting-state data was carried out using FEAT Version 6.00 within FSL (FMRIB's Software Library; www.fmrib.ox.ac.uk/fsl). The 210-volume functional dataset for each participant was realigned to compensate for small head movements using MCFLIRT (53), skull-stripped using the Brain Extraction Tool (BET) (54); spatially smoothed using a Gaussian kernel of 5 mm FWHM, intensity normalized by a single multiplicative factor, and band-pass filtered to correct for baseline drift and high frequency noise (high-pass temporal filter: Gaussian-weighted least-squares straight line fitting, with sigma = 50.0 s; low-pass temporal filter: Gaussian with sigma = 2.8 s). Functional images were registered to corresponding high-resolution T1-weighted structural images and then normalized to Montreal Neurological Institute (MNI) space using a 12-parameter transformation. Masks of white matter and cerebrospinal fluid (CSF) generated from anatomical images were applied to the functional data to extract white matter and CSF time-series. These time-series were used together with 6 motion parameters as nuisance regressors in a voxel-wise regression of the fMRI data. Data scrubbing was also performed following the method of Power et al. (55), excluding any volume in which either the value for DVARS (the root mean squared change in BOLD signal from the prior volume) or the value for framewise displacement exceeded the upper boxplot threshold (the 75th percentile plus 1.5 times the interquartile range), along with the previous volume and the 2 following volumes. Forty-Seven out of sixty-nine participants in the resting state analysis had < 33% of the volumes requiring removal, enabling inclusion in this analysis. Subjects included in the RSFC analysis did not differ from subjects excluded due to motion (*P* > 0.05 on all demographic, clinical, and OGTT-related variables). Similarly, high and low abuse groups did not differ in number of censored volumes [*t*_(47)_ = 0.403, *P* = 0.689]. Residuals of the voxel-wise regression were used in subsequent seed-based connectivity analyses.

### Resting State Functional Connectivity Analysis

A seed-based intrinsic connectivity approach was used to examine resting state functional connectivity (RSFC) with the bilateral amygdala, NAcc, and insula in three separate whole brain analyses. Seed regions were anatomically defined using probabilistic maps from the Harvard-Oxford Subcortical Structural Atlas (https://fsl.fmrib.ox.ac.uk/fsl/fslwiki/Atlases), incorporating voxels that had 25% or greater probability of being labeled as the amygdala (left: 3,456 mm^3^, right: 3,936 mm^3^), the NAcc (left: 1,088 mm^3^, right: 1,016 mm^3^) or the insula (left: 9,928 mm^3^, right: 10,080 mm^3^). Amygdala, NAcc, and insula ROIs were registered from the MNI template to each participant's preprocessed fMRI data, and the mean time-series of voxels in the right and left hemispheres for each of these regions were extracted for each participant for use as primary regressors in a GLM analysis of all other voxel time-series, resulting in individual whole-brain amygdala, NAcc, and insula RSFC maps. Talairach Daemon Labels (http://www.talairach.org) were used to find corresponding Brodmann areas.

Group differences in bilateral amygdala, bilateral NAcc, and bilateral insula RSFC were examined in separate voxel-wise *t*-tests, covarying age and sex. Subjects included in the RSFC analysis showed no difference in Full-4 IQ scores by abuse group (*P* > 0.05), so we did not include IQ as a covariate in the imaging subanalyses. Resulting statistical maps were thresholded with a height threshold of *z* > 2.3 and an extent threshold of *P* < 0.01667 (Bonferroni-corrected to control for multiple comparisons with three seeds), using Gaussian random field theory to correct for multiple comparisons. Parameter estimates (proportional to fMRI signal change) of BOLD signal response were extracted separately for each cluster and for each participant using featquery (fsl.fmrib.ox.ac.uk/fsl/fsl4.0/feat5/featquery.html) and analyzed in separate GLMs in SPSS (v.22; www.ibm.com/analytics/us/en/technology/spss/) that modeled the parameter estimate as the dependent variables and age, sex, and group as independent variables.

### Statistical Analyses

All statistical analyses were carried out using R version 3.5. *t*-Tests were used to test the association between abuse group and demographic, clinical, metabolic, and behavioral characteristics. Linear regression models were performed to test for association between abuse and OGTT-derived measures of insulin and glucose. Specifically, totaled CTQ abuse score was included in linear models as a predictor of area under the OGTT insulin curve, insulin at OGTT time points of 90 and 120 min, and glucose at OGTT 120 min. To account for various possible confounders and mediators, we included age, sex, BMI percentile, IQ, pubertal development (assessed by Tanner stage), depression severity, and total Peer Victimization Scale score as covariates in subsequent linear regression analyses.

Statistical assumptions for linear regression models were tested using regression diagnostics, including tests of normality of residuals, heteroscedasticity, linearity, and collinearity. While performing regression diagnostics, we found that insulin measures, including area under the OGTT insulin curve, were not normally distributed across all study participants. We performed log transformations and checked with the Shapiro-Wilk test that log-transformed area under the insulin curve met the normality assumption for linear regression. We also identified one subject as a statistical outlier in the association between totaled CTQ abuse score and glucose at OGTT endpoint. By reviewing Cook's distance and leverage statistics, we determined the subject's T+120 glucose level to exert undue influence on the regression, and we removed the subject from this and subsequent linear regression analyses with T+120 glucose.

Following our linear regression analysis, to determine if food-seeking behavior mediated the differences in OGTT insulin response between abuse groups, we used *t*-tests to investigate group differences in eating behavior.

Finally, with the connectivities that we identified with RSFC analysis as significantly different between abuse groups, we performed an interaction analysis between totaled abuse score and parameter estimates of BOLD signal response. These included (1) the interaction between abuse and amygdala-precuneus connectivity on fasting glucose, (2) the interaction between abuse and insula-precuneus connectivity on area under insulin curve, (3) the interaction between NAcc-prefrontal connectivity on fasting glucose, and (4) the interaction between abuse and NAcc-paracingulate gyrus connectivity on T+120 glucose.

## Results

### Demographic and Clinical Characteristics

Demographic and clinical characteristics of the 69 participants are summarized in Table 1. Fifty-nine percent of the youth in this sample were female, with diverse ethnic backgrounds (55% Caucasian), with above average IQ (102.46 ± 14.14), with an average BMI in the obese range (29.95 ± 6.17), and with moderate to severe depression severity (CDRS-R Raw Score = 54.19 ± 11.28). By grouping the participants by high or low levels of abuse (see Methods), we noted significant differences in age, IQ, and Tanner stage, so we adjusted for these variables in all subsequent linear modeling. We also examined how OGTT insulin and glucose measures varied by participants' level of abuse (Table 2). Participants in the high abuse group had significantly lower insulin levels at the 2-h time point (*P* = 0.015) and almost significantly lower areas under the insulin curve (*P* = 0.083). Glucose levels at the 2-h time point were also significantly different between the two abuse groups (*P* = 0.049), but the difference between abuse groups in areas under the glucose curve was not significant (*P* = 0.280). We found no mediating effect of food-seeking behavior in our analysis, as the results showed no significant group differences in self-reported eating behaviors or significant associations with OGTT measures.

**Table 1:** Demographic and clinical characteristics for participants overall and by abuse group.

	**All participants (*N* = 69)**	**CTQ high abuse (*N* = 35)**	**CTQ low abuse (*N* = 34)**
Age (*)	14.60 ± 2.06	15.08 ± 2.18	14.11 ± 1.83
Female sex	41 (59%)	23 (66%)	18 (53%)
Caucasian race	38 (55%)	16 (46%)	22 (65%)
Intellectual quotient (IQ) (*)	102.46 ± 14.14	98.54 ± 13.30	106.50 ± 14.03
Body mass index (BMI)	29.95 ± 6.17	31.01 ± 6.71	28.87 ± 5.44
Tanner stage (*)	3.68 ± 0.99	3.91 ± 0.92	3.44 ± 1.02
Socioeconomic status (SES) (Hollingshead Index raw score)	45.60 ± 13.78	44.43 ± 13.57	46.81 ± 14.10
Depression severity (Children's Depression Rating Scale-Revised raw score)	54.19 ± 11.28	55.91 ± 11.58	52.41 ± 10.84
Childhood trauma questionnaire (*)	39.59 ± 10.84	46.80 ± 9.97	32.18 ± 5.28
Emotional abuse (*)	10.36 ± 4.78	13.97 ± 4.02	6.65 ± 1.54
Physical abuse (*)	6.07 ± 1.86	6.74 ± 2.33	5.38 ± 0.74
Sexual abuse (*)	5.67 ± 2.30	6.31 ± 3.11	5.00 ± 0.00
Emotional neglect (*)	10.35 ± 4.25	12.17 ± 4.47	8.47 ± 3.07
Physical neglect	7.15 ± 2.17	7.60 ± 2.46	6.68 ± 1.74
Peer victimization scale	9.71 ± 7.98	10.37 ± 8.54	8.96 ± 7.39
Physical victimization	1.00 ± 2.03	1.00 ± 2.08	1.00 ± 2.00
Verbal victimization	3.96 ± 2.93	4.13 ± 2.90	3.78 ± 3.00
Social manipulation	2.53 ± 2.62	2.90 ± 2.78	2.11 ± 2.40
Attacks on property	1.89 ± 2.31	2.00 ± 2.51	1.78 ± 2.12
Three factor eating questionnaire	23.37 ± 8.39	23.76 ± 7.89	22.97 ± 8.97
Cognitive restraint	9.47 ± 4.94	9.74 ± 4.97	9.21 ± 4.97
Disinhibition scale	7.18 ± 3.69	7.38 ± 3.48	6.97 ± 3.93
Hunger	6.72 ± 3.66	6.65 ± 3.68	6.79 ± 3.69

Values reported are mean ± SD or N (%). (*) P < 0.05.

**Table 2:** Baseline metabolic characteristics from OGTT by abuse group.

	**CTQ high abuse**	**CTQ low abuse**	***P***
T+120 insulin	69.94 ± 51.06	116.68 ± 95.13	0.015
T+120 glucose	114.17 ± 33.85	128.90 ± 27.04	0.049
Area under insulin curve	9841.29 ± 5519.2	13002.35 ± 8924.0	0.083
Area under glucose curve	15691.97 ± 3501.7	16515.84 ± 2748.5	0.280
Fasting insulin	13.23 ± 8.56	14.74 ± 9.94	0.503
Fasting glucose	92.87 ± 8.64	92.33 ± 9.17	0.801
HOMA-IR	3.69 ± 2.21	4.29 ± 3.41	0.391

*T+120 insulin, insulin at time 120 min after glucose challenge; T+120 glucose, glucose at time 120 min after glucose challenge; HOMA-IR, Homeostatic Model Assessment-Insulin Resistance; CTQ, Childhood Trauma Questionnaire*.

### Abuse Group Differences in Serum Insulin and Plasma Glucose Levels During OGTT

**Figure 1:**
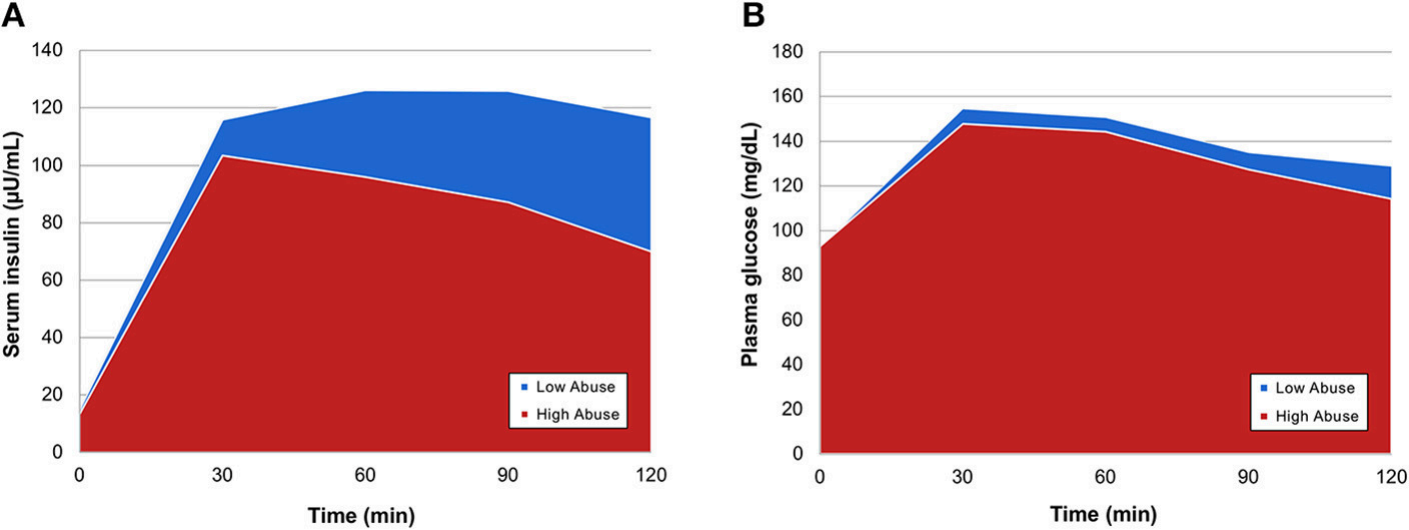
Mean OGTT curves by abuse group for **(A)** serum insulin and **(B)** plasma glucose.

In order to evaluate differences in insulin and glucose response to OGTT between the two abuse groups, we visualized each abuse group's mean serum insulin and mean plasma glucose at time point during the oral glucose tolerance test, creating metabolic response curves. A more elevated insulin response to the oral glucose challenge was observed in participants who reported a low level of abuse (Figure 1A). Insulin and glucose shapes varied between groups; mean insulin curve in the high abuse group showed earlier peak and decline, whereas the curve for the low abuse group was elevated for a sustained period. Mean glucose curves showed more similarity between the two groups (Figure 1B). Peaks for both curves were observed at the 30-min time point, followed by declines in plasma glucose.

Linear regression modeling showed that insulin and glucose response was associated with level of abuse (Table 3). Totaled CTQ abuse scores significantly correlated with log-transformed area under the insulin curve, insulin at 90 min, and glucose at OGTT endpoint. We found that age, sex, race, IQ, and depression severity did not confound or mediate the relationship between abuse and OGTT measures. On the other hand, BMI percentile and Peer Victimization Scale (PVS) total score were identified as possible negative confounders of the association between abuse and log-transformed insulin curve area (Δß = +12.5% adjusting for BMI; +29% adjusting for PVS), as well as between abuse and log-transformed T+90 insulin (Δß = +68.5% adjusting for PVS). In other words, adjusting for BMI percentile and peer victimization accentuated the significant effect of abuse on insulin response. Whereas we found significant differences in T+120 insulin between abuse groups when we treated abuse as a categorical variable, our linear modeling with totaled CTQ abuse as a continuous variable did not show correlations with insulin at OGTT endpoint.

**Table 3:** Linear regression model parameter estimates for total level of abuse in additive models predicting OGTT insulin (log-transformed) and glucose values.

	**Insulin curve area (log-transformed)**		**T+90 insulin (log-transformed)**		**T+120 insulin (log-transformed)**		**T+120 glucose**	
	**ß**	***P***	**ß**	***P***	**ß**	***P***	**ß**	***P***
CTQ total abuse score	−0.0107	0.039	−0.0090	0.175	−0.0127	0.070	−1.475	0.005
Add age, sex, Tanner stage, race, SES, IQ	−0.0112	0.060	−0.0087	0.260	−0.0115	0.146	−1.379	0.011
Add BMI percentile	−0.0126	0.034	−0.0109	0.150	−0.0126	0.118	−1.364	0.013
Add CDRS-R depression severity	−0.0131	0.030	−0.0111	0.152	−0.0132	0.106	−1.484	0.007
Add PVS total score	−0.0169	0.020	−0.0187	0.047	−0.0158	0.116	−1.252	0.049

*Values reported for the model predicting T+120 glucose are after removal of one outlier. CTQ, Childhood Trauma Questionnaire; SES, Socioeconomic Status; IQ, Intelligence Quotient; BMI, Body Mass Index; CDRS-R, Children's Depression Rating Scale-Revised; PVS, Peer Victimization Scale*.

### Abuse Group Differences in Amygdala, NAcc, and Insula RSFC

**Figure 2:**
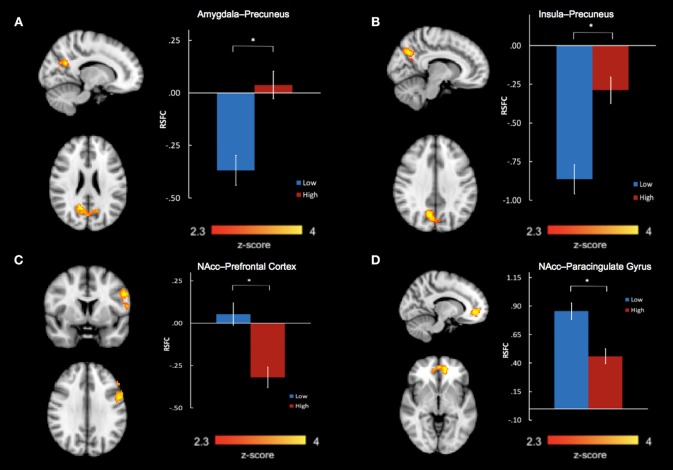
Abuse group differences in resting state functional connectivity between **(A)** bilateral amygdala and precuneus, **(B)** bilateral insula and precuneus, **(C)** bilateral nucleus accumbens and left prefrontal cortex, including the left dorsolateral prefrontal gyrus, precentral gyrus, and inferior frontal gyrus, and **(D)** bilateral nucleus accumbens and bilateral paracingulate gyri in the medial prefrontal cortex. *P < 0.01667.

Group differences in bilateral amygdala, bilateral NAcc, and bilateral insula RSFC were examined in separate voxel-wise *t*-tests, co-varying age, and sex. After correcting for multiple comparisons, we found a significant main effect of group between the bilateral amygdala and precuneus (*k* = 644 voxels, peak x/y/z MNI coordinate = 14/−58/24, *z* = 3.56, *P* = 0.000594, Brodmann areas 7, 23, 31) (Figure 2A). We also found a significant main effect of group between the bilateral insula and precuneus (*k* = 566 voxels, peak x/y/z MNI coordinate = 10/−66/34, *z* = 4.05, *P* = 0.002, Brodmann areas 7, 31) (Figure 2B). For both of these main effects, the high abuse group showed reduced negative connectivity to the precuneus compared to the low abuse group.

We also found significant group differences in connectivity between bilateral NAcc and two regions: a prefrontal cluster which included the left dorsolateral prefrontal cortex (DLPFC), precentral gyrus, and inferior frontal gyrus (IFG) (*k* = 597 voxels, peak x/y/z MNI coordinates = −54/4/30, *z* = 3.46, *P* = 0.0013, Brodmann areas 6, 9, 45, 46) and a cluster encompassing the bilateral paracingulate gyri in the medial prefrontal cortex (mPFC) (*k* = 386 voxels, peak x/y/z MNI coordinates = −12/48/−4, z = 3.89, *P* = 0.023, Brodmann areas 10, 32) (Figures 2C,D). In the high- compared to low- abuse group, there was increased negative connectivity between NAcc and the prefrontal cluster, and reduced positive connectivity between NAcc and the paracingulate gyrus cluster.

We extracted parameter estimates of BOLD signal response for these four connectivity clusters showing abuse group differences. Taking total abuse score as a continuous variable, we explored a dose relation between level of abuse and connectivity values that was significant for insula-precuneus (*P* = 0.0032, *r* = 0.421), NAcc-prefrontal (*P* = 0.0035, *r* = −0.418), and NAcc-paracingulate gyrus (*P* = 0.0046, *r* = −0.406) connectivities, and near significant for amygdala-precuneus connectivity (*P* = 0.064, *r* = 0.272).

### Abuse Level Moderates the Relation Between RSFC and OGTT Insulin and Glucose Response

**Figure 3:**
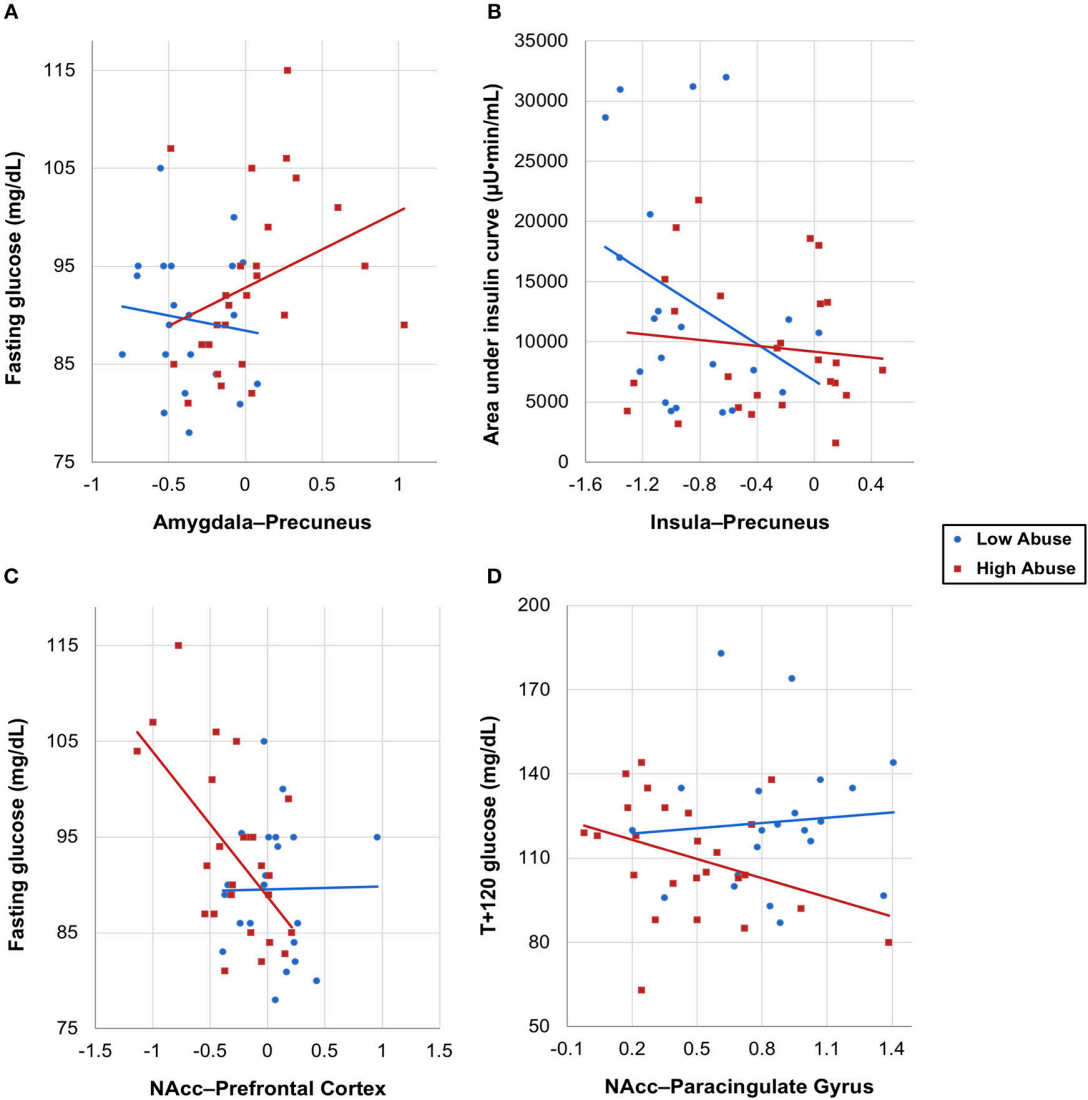
OGTT insulin and glucose measures vs. neural connectivity values by abuse group, with group-specific least-squares lines. **(A)** Fasting glucose vs. amygdala–precuneus connectivity, **(B)** area under the insulin curve vs. insula–precuneus connectivity, **(C)** fasting glucose vs. NAcc–prefrontal connectivity, **(D)** T+120 glucose vs. NAcc–paracingulate gyrus connectivity, with one outlier removed.

With extracted neural connectivity values from the four connectivity group differences shown above, we explored associations between connectivity estimates and OGTT measures of insulin and glucose. We made scatterplots with least-squares lines for each abuse group and found that the two groups had visibly different correlations between the insulin or glucose response and brain connectivity (Figure 3). The high abuse group showed correlations between OGTT measures and amygdala-precuneus, NAcc-prefrontal cortex, and NAcc-paracingulate gyrus connectivity, while the low abuse group did not (Figures 3A,C,D). On the other hand, the low abuse group showed a negative correlation between area under the insulin curve and insula-precuneus connectivity, while the high abuse group did not (Figure 3B). Adjusting for age, sex, BMI, and peer victimization, we found a significant negative correlation across all participants between log-transformed area under the insulin curve and bilateral insula-precuneus connectivity (*P* = 0.041), as well as between log-transformed T+120 insulin and bilateral amygdala-precuneus connectivity (*P* = 0.019).

Mediation analysis using connectivity values and abuse scores as covariates in linear models of OGTT measures, both study-wide and by separated abuse group, did not show significant mediation by connectivity of the effect of abuse on insulin or glucose. However, when we performed moderation analysis to see if abuse moderated the association between connectivity estimates and OGTT measures, we found that each of the four identified connectivity differences interacted with abuse in predicting OGTT measures, in line with our scatterplot findings. Cumulative CTQ abuse score as a continuous variable moderated the associations between: amygdala-precuneus connectivity and fasting glucose (interaction term *P* = 0.043), insula-precuneus connectivity and log-transformed area under the insulin curve (interaction term *P* = 0.037), and NAcc-prefrontal connectivity and fasting glucose (interaction term *P* = 0.0005) (Tables 4–6). We also found moderation by cumulative CTQ abuse score of the association between NAcc-paracingulate gyrus connectivity and T+120 glucose, trending toward significance (interaction term *P* = 0.114) (Table 7).

**Table 4:** Adjusted interaction effect of abuse and amygdala-precuneus connectivity on fasting glucose.

**Predictor**	**ß**	**SE**	***P***
(Intercept)	73.119	41.883	0.093
Age	−0.093	1.562	0.953
Male sex	3.321	3.536	0.356
Tanner stage	1.284	3.706	0.732
Non-Hispanic race	−1.835	3.624	0.617
SES	−0.278	0.146	0.068
IQ	0.056	0.135	0.683
BMI percentile	0.043	0.398	0.915
Depression severity	0.097	0.114	0.404
Peer victimization total score	0.101	0.182	0.583
CTQ total abuse score	0.492	0.277	0.088
Amygdala-precuneus connectivity	−28.857	15.630	0.076
CTQ total abuse score × Amygdala-precuneus connectivity (*)	1.439	0.675	0.043

(*) P < 0.05. Model P = 0.067, F = 2.00, adjusted R^2^ = 0.241. SES, Socioeconomic Status; IQ, Intelligence Quotient; BMI, Body Mass Index; CTQ, Childhood Trauma Questionnaire; SE, Standard Error.

**Table 5:** Adjusted interaction effect of abuse and insula-precuneus connectivity on log-transformed area under insulin curve.

**Predictor**	**ß**	**SE**	***P***
(Intercept) (*)	3.3409	1.4317	0.028
Age	−0.0118	0.0526	0.824
Male sex	−0.1271	0.1388	0.368
Tanner stage	0.0063	0.1381	0.964
Non-Hispanic race	0.0670	0.1388	0.633
SES	−0.0053	0.0057	0.366
IQ	−0.0100	0.0051	0.060
BMI percentile	0.0214	0.0130	0.113
Depression severity	0.0007	0.0045	0.878
Peer victimization total score	−0.0129	0.0073	0.088
CTQ total abuse score	0.0016	0.0141	0.908
Insula-precuneus connectivity (*)	−1.040	0.4581	0.032
CTQ total abuse score × Insula-precuneus connectivity (*)	0.0421	0.0191	0.037

(*) P < 0.05. Model P = 0.140, F = 1.644, adjusted R^2^ = 0.169. SES, Socioeconomic Status; IQ, Intelligence Quotient; BMI, Body Mass Index; CTQ, Childhood Trauma Questionnaire; SE, Standard Error.

**Table 6:** Adjusted interaction effect of abuse and NAcc-prefrontal connectivity on fasting glucose.

**Predictor**	**ß**	**SE**	***P***
(Intercept)	60.741	31.203	0.062
Age	1.971	1.127	0.092
Male sex	−0.093	2.684	0.973
Tanner stage	−2.701	2.812	0.346
Non-Hispanic race	0.877	2.840	0.760
SES (*)	−0.258	0.121	0.042
IQ	−0.033	0.102	0.752
BMI percentile	0.345	0.278	0.226
Depression severity	−0.016	0.093	0.862
Peer victimization total score	−0.011	0.146	0.941
CTQ total abuse score	−0.281	0.216	0.206
NAcc-prefrontal connectivity (*)	26.256	8.743	0.006
CTQ total abuse score × NAcc-prefrontal connectivity (*)	−1.368	0.343	0.0005

(*) P < 0.05. Model P = 0.002, F = 3.95, adjusted R^2^ = 0.482. SES, Socioeconomic Status; IQ, Intelligence Quotient; BMI, Body Mass Index; CTQ, Childhood Trauma Questionnaire; NAcc, Nucleus accumbens; SE, Standard Error.

**Table 7:** Adjusted interaction effect of abuse and NAcc-paracingulate gyrus connectivity on T+120 glucose.

**Predictor**	**ß**	**SE**	***P***
(Intercept) (*)	261.149	119.17	0.038
Age	−1.806	4.061	0.660
Male sex	10.813	9.726	0.277
Tanner stage	−3.235	9.819	0.745
Non-Hispanic race	18.523	9.947	0.074
SES (*)	−0.913	0.429	0.043
IQ	−0.203	0.354	0.571
BMI percentile	−1.091	0.945	0.259
Depression severity	0.572	0.335	0.100
Peer victimization total score	0.051	0.570	0.929
CTQ total abuse score	0.409	1.471	0.784
NAcc-paracingulate gyrus connectivity	69.102	44.543	0.133
CTQ total abuse score × NAcc-paracingulate gyrus connectivity	−2.982	1.820	0.114

(*) P < 0.05. Model P = 0.116, F = 1.75, adjusted R^2^ = 0.1951. One outlier removed. SES; Socioeconomic Status; IQ, Intelligence Quotient; BMI, Body Mass Index; CTQ, Childhood Trauma Questionnaire; NAcc, Nucleus accumbens; SE, Standard Error.

## Discussion

Our findings suggest that depressed and overweight youth with high abuse exposure have different neural and endocrine characteristics compared to low abuse-exposed counterparts. Further, levels of abuse seem to be related to levels of hyperglycemia and insulin resistance. However, contrary to our hypotheses, lower levels of abuse were associated with post-glucose challenge hyperglycemia, hyperinsulinemia, and a persistently elevated glucose response. Neurally, the low abuse group showed more negative bilateral insula-precuneus connectivity than the high abuse group. More negative insula-precuneus connectivity was associated with higher area under the insulin curve and hyperinsulinemia. In contrast, overweight and depressed youth with high abuse showed dysfunctional connectivities between the amygdala-precuneus, NAcc-paracingulate gyrus, and NAcc-prefrontal networks at rest. Their levels of connectivity in these networks correlated with fasting and post-glucose challenge glucose levels. These unexpected results suggest possible compensatory or group-independent mechanisms that may be unique to our cohort and merit replication with larger sample sizes and broader comparison groups.

Although brain connectivity varied between abuse groups, glucose response to OGTT varied independently of abuse. The insignificant difference in glucose response between abuse groups was not unexpected, as previous literature has suggested the same. Specifically, in youth with insulin resistance, blood glucose levels may appear normal, as pancreatic beta-cell reserves may mount compensatory hyperinsulinemic responses (39). However, the significant difference in insulin response between high and low abuse groups was surprising. We propose two possible explanations for this finding. First, highly stressed children may experience insulin deficiency due to beta cell dysfunction as a result of their exposure to abuse. This is supported by previous studies that showed early life stress increases the risk of type 1 diabetes (56). It also is supported potentially by a central brain mechanism, as illustrated by our neuroimaging results which link aberrant resting state brain connectivity with lower insulin and higher glucose responses. However, the average glucose curve for our high abuse group was not observed to be more elevated than that of our low abuse group, as would be expected. An alternative explanation may be that hyperinsulinemia in the high abuse group is a transient compensatory response to high stress, arising through sympathetic overdrive (57, 58). This transitory hypersensitivity may be supported by the slightly lower glucose curve for our highly abused participants. In either case, this finding suggests an early form of metabolic dysfunction in youth in the high abuse group that may be evolving but not yet fully realized.

The group differences in resting state brain connectivity demonstrate the impact of abuse on neural function in depressed and obese youth, specifically in correlated brain regions that form a network to process emotion and self-perception (59), as well as motivation, reward, and executive control over emotion and reward (60). In our investigation of the amygdala and insula as common denominator regions in depression and obesity, our data implicate the precuneus for both, highlighting the importance of self-referential thinking in high abuse-exposed youth. We observed less negative amygdala-precuneus connectivity in high abuse compared to low abuse youth with depression and obesity, which may result in impaired emotion processing and regulation (61, 62), problems with self-reflection (63, 64), and lower self-esteem in these youth (65). Indeed, less negative amygdala-precuneus connectivity has been described in adults with a history of childhood trauma (66). Our findings also showed decreased negative (toward more positive) connectivity between the insula and precuneus in the high abuse compared to the low abuse group. Childhood abuse has similarly been shown to be associated with an increase toward more positive connectivities in both the precuneus and insula within each of their neural networks (67). In the context of maltreatment, the insula has been reported to play an additive role with the precuneus toward dysfunctional self-awareness and emotional processing (68). Thus, our findings provide a neural network basis for the emotion dysregulation and negative self-concept commonly characterized in youth with depression and obesity (69, 70), which our data suggest may be compounded by high levels of abuse.

Emerging literature suggests that altered self-awareness due to dysfunctionally increased precuneus connectivity may be observed in addicted populations (71). Given the conditioned response to food cues that forms the basis of addictive behaviors in youth with co-occurring depression and obesity, we investigated regions correlated at rest within the brain's reward network. The NAcc is a critical region in the reward network, sending dopaminergic neurons to other components of the network (19). Decreased inhibitory control over food-seeking behaviors is a central feature of obesity, which is frequently represented by positive intrinsic7 connectivity between reward networks and the prefrontal cortex (72–74). Although we did not find differences in eating behavior between abuse groups, we did find that the high abuse group had increased *negative* connectivity between the NAcc and key regulatory subregions in the left prefrontal cortex, including the left DLPFC, IFG, and precentral gyrus. The prefrontal cortex is important for executive control of emotion and reward, processing behavioral rules in reward (75). Greater negative connectivity between NAcc and portions of the prefrontal cortex including the DLPFC has been reported in individuals with alcohol abuse (67), depression (76), and with exposure or vulnerability to abuse across species (77–79), implicating this aberrant connectivity pattern in addiction and stress. Recent manipulation of NAcc-prefrontal network connectivity through transcranial magnetic stimulation to treat depression (80) and through dietary intervention to treat obese individuals (81) illustrates the robust replicability and utility of this neural system as a treatment target. Similarly, the high abuse group also showed reduced negative connectivity between the NAcc and paracingulate gyrus compared to the low abuse group. The paracingulate gyrus has been shown to be less activated in major depressive disorder during anticipation of monetary rewards (82), just as the subgenual portions of the anterior cingulate is less connected to the default mode network in abuse-exposed youth with higher levels of behavioral activation system sensitivity (83). Interventions that bolster behavioral activation may protect against the development of stress-related disorders by modifying the central neural circuit implicated in rumination. Indeed, the disruption between the NAcc and paracingulate gyrus in the high abuse group supports that abuse is associated with atypical regulation of the reward network, possibly through a decoupling mechanism.

We speculate that this decoupling of the frontostriatal network in high abuse youth may influence dopamine release in the VTA, which may further influence insulin sensitivity toward developmental adaptation. Studies on non-obese adults demonstrate that acute dopamine depletion leads to increased fasting insulin levels in diabetic and healthy individuals (84, 85). Consistently, increasing striatal dopamine levels by deep brain electrical stimulation reduces fasting insulin levels (85), suggesting the apparent link between striatal function and systemic insulin sensitivity, as demonstrated in our sample. Moreover, children who have experienced physical or sexual abuse exhibit higher striatal dopamine synthesis when they reach adolescence and young adulthood (86), and reduced dopamine levels have not been observed in depressed adults. Therefore, the lower insulin levels in our high abuse group may be a developmental adaption related to decreased regulation of NAcc connectivity, leading to increased striatal dopamine levels that improve insulin sensitivity. In other words, high abuse youth show better metabolic functioning, suggesting potentially better NAcc and dopamine functioning, which requires less prefrontal connectivity and consequent regulation. Alternatively, inhibition of the prefrontal cortex by high abuse may lead to a lack of regulation of NAcc connectivity, requiring greater levels of striatal dopamine synthesis. Regardless, this unique finding is confirmed by our moderation analysis, which yields correlations between NAcc connectivity and OGTT measures that are significant for high but not low abused youth.

We had hypothesized that deregulation of reward network connectivity may relate to a deregulation of eating behavior that mediates insulin resistance in youth. A preclinical mouse model of hyperinsulinemia in which insulin does not induce an expected synaptic depression of ventral tegmental dopamine neuron activity, raises one possibility that reward circuit function may be related to the disruption in typical insulin-glucose function, that may, in turn, be related to increased feeding behavior (87). However, our data did not show OGTT response differences in high and low abuse groups due to differences in eating behavior. Our finding is more consistent with the mixed and non-standardized evidence in humans regarding how dopamine release, endogenous dopamine levels, and dopamine D2 receptor expression in the NAcc and VTA are related to eating behavior (88–90).

We propose an alternative hypothesis that abuse may effect change on signaling between the brain and the pancreas. If abuse were to impact an intermediary between brain connectivity and insulin production, then it would also cause downstream effects on the neural and pancreatic targets of that intermediary. One potential intermediary is cortisol, which has been shown to be lowered by childhood abuse (91, 92). Cortisol is a glucocorticoid secreted by the hypothalamic-pituitary-adrenal (HPA) axis and has been shown to influence insulin levels and to correlate with amygdala resting state functional connectivity (93–95). Similarly, inflammatory mediators influence frontolimbic and frontostriatal circuits in such a way as to predispose individuals toward self-medicating behaviors such as consumption of high fat diets (96). Further studies are warranted to investigate the intriguing roles of cortisol and inflammatory markers in mediating relations among abuse, insulin secretion and sensitivity, and brain connectivity.

We should note four study limitations. First, we had a modest sample size for the multilevel assessments conducted. For our sample of youth with combined depression and obesity, and with varying levels of abuse histories, there were no referent effect sizes for group differences in resting state connectivity. Still, a comprehensive neurobiological assessment of the magnitude of impact of abuse on insulin resistance in youth with co-occurring depression and obesity has not been presented in the literature before, and is an important hypothesis-generating starting point in a growing population of youth. Second, our cross-sectional design without a typically developing comparison group made it difficult to assess degree of insulin resistance on OGTT, and to interpret our functional connectivity findings as markers of neural vulnerability or resilience. Comparing youth in our study to non-obese, non-depressed, or non-obese and depressed groups may more fully capture the range, level, and degree of insulin resistance and reward network dysregulation in these youth. Prior studies of non-obese youth with depression have shown increased amygdala-precuneus intrinsic connectivity compared to controls (97). In contrast, non-depressed youth with obesity have shown reduced insula-anterior cingulate and middle-temporal gyrus-cuneus intrinsic connectivity compared to controls (98). However, to our knowledge, no study has examined varying levels of abuse history in youth with both depression and obesity. Third, though our study focused on childhood abuse as measured by the CTQ, there may be other sources of early life stress that may contribute to the findings presented but were beyond the scope of the current study. Our results suggest that peer victimization may moderate the relation between abuse and insulin response after covarying for socioeconomic status. Indeed, prior studies report that both overweight (99) and low social status (100) children are more likely to be bullied. Further, both obesity and reduced social status have been associated with reduced dopamine D2 receptor expression in adults (101–104). Future studies that look at the neural and endocrine correlates of peer victimization—covarying for both socioeconomic status and childhood abuse—may provide further insights into the effects of early life stress. Finally, though our study focused on insulin resistance, many endocrine abnormalities may coincide with obesity. Our ongoing analyses of these other endocrine contributors were beyond the scope of the aims of this study.

Our results suggest a unique interaction between abuse, depression, and obesity in youth in terms of neural connectivity patterns and metabolic function. By characterizing the neural and endocrine impact of abuse in youth with depression and obesity, our findings can create better profiles for children with psychiatric disorders based on histories of early childhood stress exposure. Identifying these profiles can promote early intervention (105) and possibly interrupt trajectories toward chronic conditions (106). Future studies to further characterize high and low abuse groups will include longitudinal analysis of insulin resistance and changes in brain connectivity over the course of development. These prospective studies will help determine whether exposure to early childhood adversity predicts the expected progression of insulin resistance and depression outcomes. In addition, further research differentiating peer victimization from abuse derived from family settings can refine these neural and endocrine profiles by delineating specific early life stress factors and their impact.

## Author Contributions

MS conceptualized and received funding for the study and executed the study protocol. MS, KS, SA, BB, and AG contributed to the analyses and writing of the manuscript including the figures and tables. SL contributed to the analysis of imaging data. KW and NR assisted with the analysis of data and contributed to the final draft of the manuscript.

### Conflict of Interest Statement

MS receives research support from Stanford's Maternal Child Health Research Institute, National Insitute of Mental Health, National Institute of Aging, Neuronetics, Johnson and Johnson, and the Brain and Behavior Foundation. She is on the advisory board for Sunovion and Google X. NR receives research support from the National Institute of Aging. The remaining authors declare that the research was conducted in the absence of any commercial or financial relationships that could be construed as a potential conflict of interest.
